# Evolution of *PHAS* loci in the young spike of Allohexaploid wheat

**DOI:** 10.1186/s12864-020-6582-4

**Published:** 2020-03-04

**Authors:** Rongzhi Zhang, Siyuan Huang, Shiming Li, Guoqi Song, Yulian Li, Wei Li, Jihu Li, Jie Gao, Tiantian Gu, Dandan Li, Shujuan Zhang, Genying Li

**Affiliations:** 1 Crop Research Institute, Shandong Academy of Agricultural Sciences, Jinan, 250100 Shandong China; 2 Key Laboratory of Wheat Biology and Genetic Improvement on North Yellow and Huai River Valley, Ministry of Agriculture, Jinan, 250100 Shandong China; 3 National Engineering Laboratory for Wheat and Maize, Jinan, 250100 Shandong China; 4 BGI Institute of Applied Agriculture, BGI-Shenzhen, Shenzhen, 518120 China

**Keywords:** PhasiRNAs, *PHAS*, MicroRNAs, Evolution, Young spike, Wheat

## Abstract

**Background:**

PhasiRNAs (phased secondary siRNAs) play important regulatory roles in the development processes and biotic or abiotic stresses in plants. Some of phasiRNAs involve in the reproductive development in grasses, which include two categories, 21-nt (nucleotide) and 24-nt phasiRNAs. They are triggered by miR2118 and miR2275 respectively, in premeiotic and meiotic anthers of rice, maize and other grass species. Wheat (*Triticum aestivum*) with three closely related subgenomes (subA, subB and subD), is a model of allopolyploid in plants. Knowledge about the role of phasiRNAs in the inflorescence development of wheat is absent until now, and the evolution of *PHAS* loci in polyploid plants is also unavailable.

**Results:**

Using 261 small RNA expression datasets from various tissues, a batch of *PHAS* (phasiRNA precursors) loci were identified in the young spike of wheat, most of which were regulated by miR2118 and miR2275 in their target site regions. Dissection of *PHAS* and their trigger miRNAs among the diploid (AA and DD), tetraploid (AABB) and hexaploid (AABBDD) genomes of *Triticum* indicated that distribution of *PHAS* loci were dominant randomly in local chromosomes, while miR2118 was dominant only in the subB genome. The diversity of *PHAS* loci in the three subgenomes of wheat and their progenitor genomes (AA, DD and AABB) suggested that they originated or diverged at least before the occurrence of the tetraploid AABB genome. The positive correlation between the *PHAS* loci or the trigger miRNAs and the ploidy of genome indicated the expansion of genome was the major drive force for the increase of *PHAS* loci and their trigger miRNAs in *Triticum*. In addition, the expression profiles of the *PHAS* transcripts suggested they responded to abiotic stresses such as cold stress in wheat.

**Conclusions:**

Altogether, non-coding phasiRNAs are conserved transcriptional regulators that display quick plasticity in *Triticum* genome. They may be involved in reproductive development and abiotic stress in wheat. It could be referred to molecular research on male reproductive development in *Triticum*.

## Background

There is a particular class of small RNAs generated in 21- or 24-nt (nucleotide) intervals with a ‘head-to-tail’ pattern from their precursor transcripts, which are called phased, secondary, small interfering RNAs (phasiRNAs) [1–3]. PhasiRNAs have been found both in animals and plants. The genes encoding PIWI-interacting RNAs (piRNAs), which are a type of phasiRNAs, are required for producing mature sperm in animals [4]. In plants, phased siRNAs play a series of roles in abiotic and biotic stresses [5, 6], seed germination [7] and reproductive development [3, 8–10]. Trans-acting siRNAs (ta-siRNAs) are a special class of phasiRNAs generated from *TAS* precursors (noncoding or coding transcripts), which silence targets in *trans*. In addition, the regulation of disease resistance genes with the *NB-LRR* (nucleotide-binding sites and leucine-rich repeat) domains by miRNAs has mostly been characterized in the ETI (effector-triggered immunity) pathway of plants [11]. MiRNAs [12–14] can also trigger 21-nt phasiRNA generation from *NB-LRR* transcripts, and most of them are species-specific. Some miRNA affects seed germination by generating phased siRNAs and modulating abscisic acid/gibberellin signaling in wheat [7].

In reproductive tissues, phasiRNAs are active in anther development of both eudicots and monocots. In grasses, there are two pathways that yield abundant phasiRNAs, which are associated with meiosis [3, 8]. MiR2118 triggers one class of 21-nt phasiRNAs in premeiotic anther development, while miR2275 triggers another class of 24-nt phasiRNAs [8]. In eudicots, 24-nt phasiRNAs are also present in the anther or pollen development triggered by either miR2275 or not [15]. This finding indicated the ancient origin of phasiRNAs and their regulatory mechanism. In general, 5′-capped and polyadenylated noncoding *PHAS* RNAs transcripted by RNA polymerase II could generate 21- or 24-nt phasiRNAs, which mediates by miR2118 or miR2275, respectively. Then, the 3′ mRNA fragments are converted into double-strand RNAs by RNA-dependent RNA polymerase 6 (RDR6), which are processed by DCL4 (dice like 4) or DCL5 (also named DCL3b) to yield 21- or 24-nt phasiRNAs [16]. Mutations in *DCL4*, *DCL5* and *RDR6* in rice affect the generation of 21- or 24-nt phasiRNAs [16, 17]. These phasiRNAs are subsequently loaded into AGOs to function their regulatory roles. In rice, MEL1 (also named OsAGO5c) preferentially binds to 21-nt phasiRNAs [18]. ZmAGO18b is enriched in the tapetum and germinal cells, and its expression pattern is similar to that of 24-nt phasiRNAs [8].

PhasiRNAs have been identified in a number of flowering plants in eudicots and monocots. In rice inflorescence, 828 and 35 of 21- and 24-*PHAS* were identified by Johnson et al [3], which could produce 21- and 24-nt phasiRNAs, respectively. In addition, 1136 and 1540 of 21-*PHAS* were identified in 93–11 and *Nipponbare*, respectively [9]*.* 70 and 34 of 24-*PHAS* were identified in 93–11 and *Nipponbare* by Song et al [16], respectively. In maize, 463 and 176 of 21-*PHAS* and 24-*PHAS* loci were identified [8]. In the flower of litchi, 178 of 21-*PHAS* loci were identified [15]. These generation and regulation mechanisms are very conserved in grasses.

Numerous phasiRNAs are involved in the anther development process, which indicates that they may play a key role in normal anther development [8]. Until now, few phasiRNAs involved in developing inflorescence have been characterized in *Triticum*. Only miR9863 and miR9678 were characterized to mediate the generation of phasiRNAs in biotic stress [6] and seed germination [7], respectively. PhasiRNAs have been increasingly recognized as an important class of regulatory RNAs in several plant species. However, much remained unknown in the wheat genome regarding sequence information and expression levels of phasiRNAs, and the evolutionary path of phasiRNAs among their progenitors and the modern hexaploid wheat is still unclear. The large and complex genomes, including wheat and their progenitors (AA, DD and AABB genomes), are all available at the present. Furthermore, the number of small RNA datasets from high-throughput sequencing deposited in public databases, is increasing. And these datasets contain various developmental and stress samples of wheat. These efforts make it possible to systematically identify the phasiRNAs in wheat.

Here, using small RNA datasets in wheat, we investigated the *PHAS* loci in various tissues, such as leaves, roots, flag leaves, young spikes, and grain. The evolution of these *PHAS* loci in *Triticum* species indicated that their independently dynamic evolution was accompanied by their regulators miR2118 and miR2275. This study will be referred for the research of anther development in wheat.

## Results

### Identification of 21- and 24-*PHAS* in wheat

**Fig. 1:**
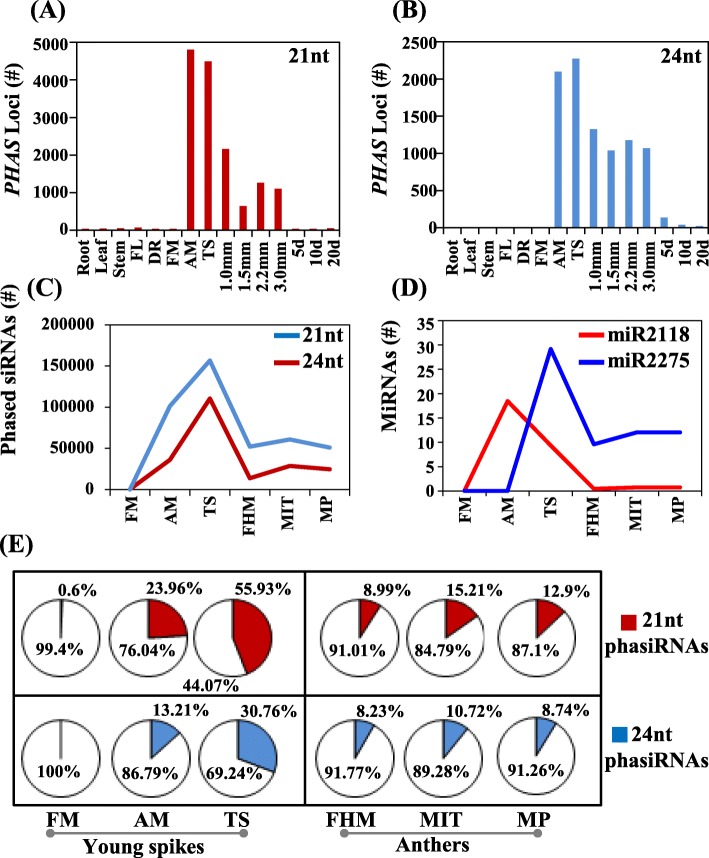
The number of 21- **a** or 24-*PHAS* loci **b** in roots, leaves, stems, flag leaves (FLs), young spikes (DR, FM, AM, TS), anthers with lengths of 1.0, 1.5, 2.2 and 3.0 mm, and grain with 5, 10 and 20 days after flowering. **c** The expression level (TPM) of 21-nt phasiRNAs (blue lines) and 24-nt phasiRNAs (red lines). The expression level (TPM) of miR2118 and miR2275 in the FM, AM, TS, FHM, MIT, and MP stages. **e** The proportion of 21-nt phasiRNAs (red color) and 24-nt phasiRNAs (blue color) in the total 21- and 24-nt siRNAs, respectively

To identify the *PHAS* loci in wheat, we downloaded 261 small RNA datasets from the GEO database (Table 1), which included 12 seedling samples, 128 leaf samples, 12 root samples, one stem sample, one shoot sample, 29 young spike samples, two anther samples, one embryo sample, 17 spikelet samples, 12 rachis samples, 19 grain samples, 20 seed samples, 6 callus samples, and one mixed tissue sample. By comparing these small RNAs to the wheat genome using the Shortstack package [19] following the flowchart as shown in Supplementary Fig. 1, a batch of *PHAS* (phasiRNA precursors) loci were identified with phased scores greater than 15, 20, 25 or 30 (Supplementary Table 1). In the reproductive tissues such as young spikes and anthers (Fig. 1a-b), abundant 21- and 24-*PHAS* were identified. However, In the leaf, stem, root, spikelet, seed, callus, etc., few *PHAS* were identified (Supplementary Table 1 && Supplementary Fig. 2). The abundance of phasiRNAs was not correlated with the total reads of the small RNA samples, but it was highly related to the types of tissues (Supplementary Fig. 2).

**Table 1:** The tissues of small RNA datasets used to identify the *PHAS* loci in wheat

Tissues	Number of samples
Seedling	12
Leaf	128
Root	12
Stem	1
Shoot	1
Young Spike	29
Anther	2
Embryo	1
Spikelet	17
Rachis	12
Grain	19
Seed	20
Callus	6
Mixed tissues	1
Total	261

In the downloaded small RNA samples of wheat, there were a group of datasets, which included the important development stage of young spike [20], i.e., DR stage (double-ridge stage) at the initiation of spike formation and spikelet development; FM stage, the stage of appearance of the floret meristems (FMs) glume primordia, and lemma primordia; AM stage (anther primordia stage), stamen and pistil primordia emerged from FMs with visible anther primordia for some florets; TS stage (tetrads stage), young florets began to differentiate with immature anthers and unelongated pistils, and the pollen mother cells completed meiosis to form the tetrads at this stage. In the DR and FM stages of young spike tissues, there were few *PHAS* loci, while in the AM and TS stages, there were abundant 21- and 24-*PHAS*. For example, ~ 3600 and ~ 4000 of the 21-*PHAS* loci with phased scores greater than 30, and 1200 and 1000 of the 24-*PHAS* loci with phased scores greater than 30 were identified in AM (SRR3690677 and SRR3690678) and TS (SRR3690679 and SRR3690680) samples, respectively (Supplementary Table 1). The number of 21-*PHAS* loci in anthers with lengths of 1.0 mm were more than in those with lengths of 1.5, 2.2 and 3.0 mm, while for the 24-*PHAS*, the number of *PHAS* loci were very similar among the different length of anther (Fig. 1a-b). According to morphological development and stage determination of young spike and anther in wheat [21], six small RNA datasets were selected for further study that represented the early and later anther development stages. The early anther development stage included the floret meristem (FM, SRR5460941 and SRR5460949), anther primordia (AM, SRR5460967 and SRR5460972) and tetrad stages (TS, SRR5461176 and SRR5461177), and the later anther development stage included free haploid microspores (FHM, SRR449365 with 1.5 mm anther), mitosis (MIT, SRR449366 with 2.2 mm anther), and mature pollen (MP, SRR449367 with 3.0 mm anther). In the next section, these *PHAS* with score more than 30 were selected to do the further analysis.

During wheat inflorescence development, the expression level of 21-nt phasiRNAs at one particular *PHAS* locus may vary at different stages. In the FM stage, there were few 21-nt phasiRNAs (300 TPM (transcripts per million)) with 0.6% out of the total 21-nt small RNAs, while in AM, a sharp increase (36,054 TPM) was observed, comprising 23.96% out of the total. In the TS stage, the expression level continued to increase with 11,410 TPM of 21-nt phasiRNAs (44.07% out of the totals) (Fig. 1c & e). For the 24-nt phasiRNAs, the tendency of the proportion was similar to that of the 21-nt phasiRNAs in the three stages. The proportions of 24-nt phasiRNAs out of the total 24-nt siRNAs were 0, 13.21% (101,277 TPM), and 30.76% (156,595 TPM) for the FM, AM, and TS stages, respectively (Fig. 1c & e). This indicated that the 21- or 24-nt phasiRNAs and 21- or 24-*PHAS* loci were present in the AM stage and peaked in the TS stage. For the later anther development stage, the 21- and 24-nt phasiRNAs occurred in the FHM stage with proportions of 8.99% (13,701 TPM) and 8.23% (52,030 TPM), peaked in the MIT stage with proportions of 15.21% (28,633 TPM) and 10.72% (60,758 TPM), and then decreased with proportions of 12.9% (24,562 TPM) and 8.74% (50,908 TPM), respectively (Fig. 1c & e). The proportions of 21- and 24-nt phasiRNAs in the three later anther development stage, were much lower than that of the AM or TS stage.

The synthesis of phasiRNAs in monocot reproductive tissues requires both *PHAS* precursors and their initiated miRNAs, such as miR2118 and miR2275 [16, 17]. The expression level of miRNAs was concertedly related to the synthesis of the phasiRNAs and *PHAS* loci. For 21- and 24-*PHAS*, the *PHAS* loci peaked both in AM and TS, and then rapidly decreased in the later anther development stage (Fig. 1a-b). Both 21- and 24-nt phasiRNAs were expressed in AM, peaked only in TS and rapidly descended in the later anther development stage (Fig. 1c & e). We then investigated the concert of the three elements including *PHAS*, phasiRNAs and their regulated miRNAs at the transcriptome level. The expression of miR2118 peaked in AM, which was similar to that of 21-*PHAS*, but occurred before the burst of 21-nt phasiRNAs. Then, the expression of miR2118 decreased in TS and disappeared in the later anther development stage. The expression of miR2275 peaked in TS, which was before that of 24-nt phasiRNAs but the same as that of 24-*PHAS.* Then, the expression of miR2275, 24-nt phasiRNAs and 24-*PHAS* rapidly decreased in the later anther development stage (Fig. 1a-e). The expression of miR2275 was higher than that of miR2118 in the later anther development stage, which may be associate with the higher expression of 24-nt phasiRNAs than 21-nt phasiRNAs in these stages (Fig. 1a-e). Together, the expression of the 21- and 24-nt phasiRNAs and their trigger miRNAs both burst in the early anther development stage.

### The distribution of *PHAS* loci in the wheat genome

To investigate the genome distribution of *PHAS* in wheat, we used the Circos package to show the number of *PHAS* loci sliding the chromosome in window sizes of 500 kb. Here, we selected these small RNA datasets with abundant phasiRNAs in AM, TS, FHM, MIT and MP for further study. In the five developmental stages, the distribution of genome elements such as repeat sequences, gene body and intergenic regions were very similar in both 21- and 24-*PHAS* (Supplementary Fig. 3). Few *PHAS* were located in the gene body or repeat sequence regions in the genome. Only 1~2% of these *PHAS* loci were distributed in gene body regions, and 12~21% of them were distributed in repeat sequence regions. In contrast, most of them (78~87%) were located in the intergenic regions. This result indicates that most *PHAS* loci may have independent transcript units that are not juxtaposed with the repeat sequences or coding genes.

According to the genome locations, we respectively merged all of the 21- and 24-*PHAS* in these samples derived from five inflorescence development stages. In total, there were 4850 and 3600 of unique 21- and 24-*PHAS* in these samples, respectively (Supplementary Fig. 4A-B), most of which were common. In total, 94.93% (2042 out of 2151), 53.14% (2385 out of 4488), 98.61% (637 out of 646), 94.85% (1263 out 1198) and 95.91% (1056 out of 1101) of 21-*PHAS* (Supplementary Fig. 4A), and 49.87% (993 out of 1991), 69.09% (1571 out of 2274), 98.26% (1019 out of 1037), 98.38% (1157 out of 1176) and 98.79% (1057 out of 1070) of 24-*PHAS* (Supplementary Fig. 4B), were overlapped each other in at least two samples in AM, TS, FHM, MIT, and MP, respectively. The low number of common 21-*PHAS* in TS and 24-*PHAS* in AM indicated that there may be more tissue-specific *PHAS* loci in these two development stages, which may be associated with the transition of the development stage from floret meristem to meiosis.

**Fig. 2:**
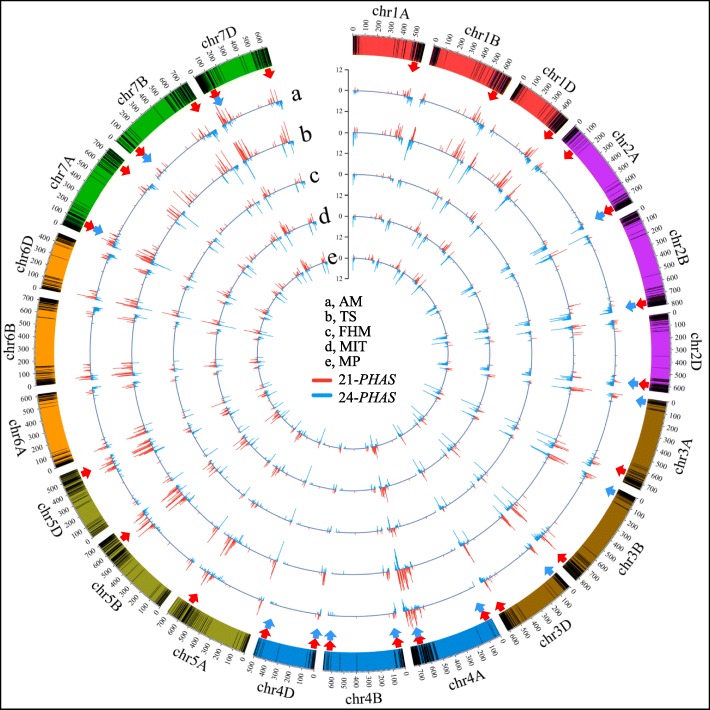
Density distribution of 21- (red lines) and 24-*PHAS* loci (blue lines) in the young spike samples. The black lines in the chromosome represent the *PHAS* loci. The peaks in circles a-e indicate the number of *PHAS* loci in each 500 kb region across each chromosome in AM, TS, FHM, MIT and MP, respectively. The red and blue arrows represent the biased distribution of 21- and 24-*PHAS* among the three subgenomes, respectively

**Fig. 3:**
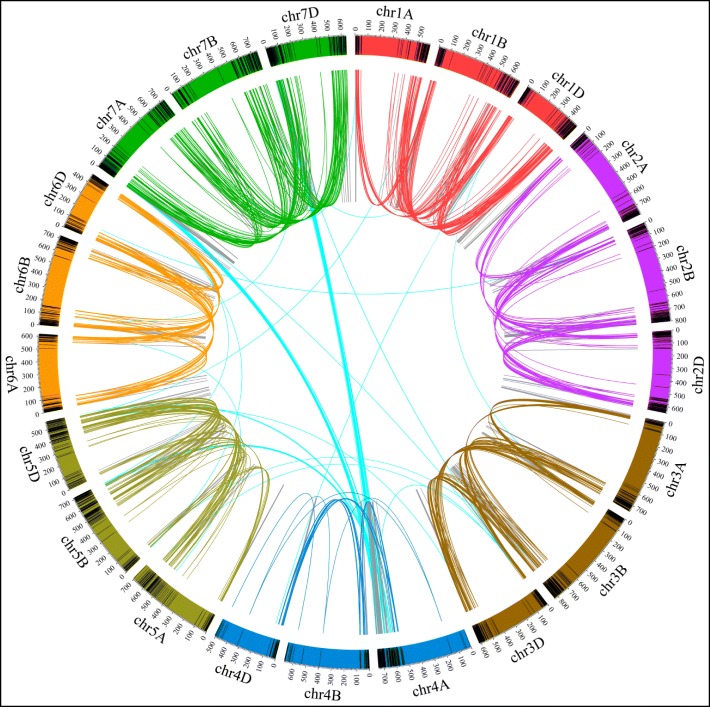
The duplication relationship among the three homoeologous chromosomes. The homoeologous *PHAS* loci are linked by lines with the same color. The black bar represents the *PHAS* loci in each chromosome

These merged unique *PHAS* were plotted on the chromosome rainbows with black lines (Fig. 2). Most of the *PHAS* loci were located at the end of the chromosomes, i.e. telomere regions. In most regions, the peaks of the 21-*PHAS* (red lines in Fig. 2) were higher than those of the 24-*PHAS* (blue lines in Fig. 2) in the representative tissues. Most of the peaks in both 21- and 24-*PHAS* were similar among the subA, subB and subD genomes in each sample. However, some peaks of 21- and 24-*PHAS* in local chromosomes were preferred among the three subgenomes in each sample (red and blue arrows in Fig. 2 and Fig. 3**).**

### The genome plasticity of *PHAS* in Allohexaploid wheat

Polyploidization is followed by genome partitioning or fractionation processes, i.e. a genome-wide diploidization, in which one or the other gene duplicate is lost. During this process, differently functional protein-coding genes have been shown to behave differently. Transcription factors or regulators are often retained as duplicated copies following whole genome duplications (WGDs), whereas others are progressively deleted back to a single copy (singleton) state [22–24]. For allohexaploid wheat (AABBDD, *Triticum aestivum*, 2n = 6x = 42), high sequence similarity and structural conservation are retained with limited gene loss after polyploidization [25]. However, at the transcript level, cell type- and stage-dependent genome dominance was observed in the local chromosome regions [26]. Studies of the roles of *PHAS* loci as a category of noncoding genes in the partitioning process of allohexaploid wheat have still not been performed. To investigate the location of *PHAS* in the subA, subB and subD of allohexaploid wheat, we performed a blast search against all genomes to identify the relationship of *PHAS* among the three subgenomes. With 80% identity and 80% matched sequence length, only 2.27%~ 6.40% of *PHAS* in the five samples retained the triplet copies in the three subgenomes, 11.11%~ 17.51% of *PHAS* retained the duplet copies in any two subgenomes, and 76.09%~ 86.22% of *PHAS* retained only singleton in any one subgenome (Supplementary Fig. 5). The homoeologous relationship is shown in Fig. 3 with the same color link lines in the homoeologous chromosomes. There were also some translocated homoeologs (the cyan links in Fig. 3). For example, some *PHAS* in chr4A were homologous to those in chr5B/D and chr7A/D. These data showed that only partial *PHAS* retained the triple or duplet homoeologs, and most *PHAS* only possessed the singleton copy.

**Fig. 4:**
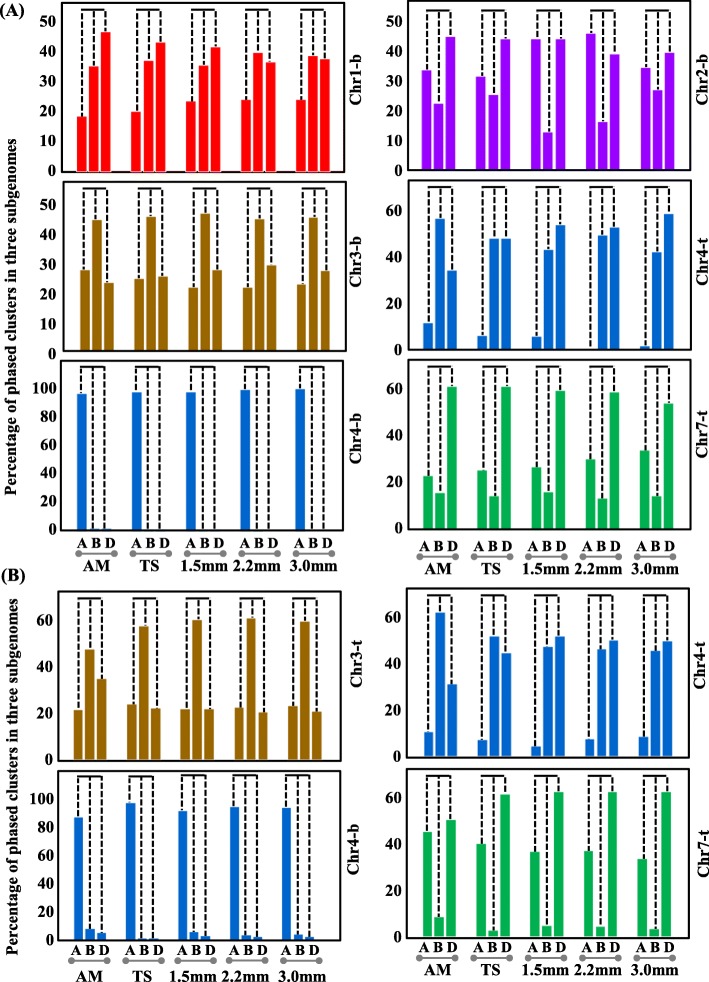
The biased distribution of 21- **a** and 24-*PHAS* loci **b** in each subgenome in AM, TS, FHM, MIT and MP. **a**, **b** and **d** indicate subA, subB and subD, respectively. “-t” and “-b” indicate the top of chromosome and bottom of chromosome from the centromere regions

To investigate the subgenome distribution of *PHAS* loci in allohexaploid wheat, the total number of *PHAS* loci in each sub-chromosome was calculated, and there were no biased in each subA, subB and subD chromosomes. However, for the local chromosomes, there were some bias distribution in the local subA, subB and subD genomes. For 21-*PHAS* loci, in the bottom chromosomes of chr1, chr2, chr3 and chr4, and in the top and bottom chromosomes of chr4 and chr7, the *PHAS* loci were biased located in the chromosomes, but the tendency of preference was different, in either the top or bottom of one chromosome (Fig. 2 (the red arrow regions) and Fig. 4a). In chr1-b (b, bottom of the chromosome), significantly less 21-*PHAS* were located in the subA genome than in the subB and subD genomes (Fisher’s exact test, *P-value* < 0.05). In chr2-b, the number of 21-*PHAS* was significantly less in subB than in subA and subD (*P-value* < 0.05). In chr3-b, subB became the dominant genome with significantly more 21-*PHAS* than the subA and subD genomes (*P-value* < 0.05). In chr4-t (top of the chromosome), the number of 21-*PHAS* was less in subA than in subB and subD (*P-value* < 1.0e-5), while in chr4-b, the subA genome possessed numerous 21-*PHAS*, significantly more than the other subgenomes (*P-value* < 2.2e-16). In chr7-t, subB possessed less of the phased loci, next to the subA genome, and the subD possessed much more 21-*PHAS* than subA and subB (*P-value* < 0.001).

For 24-*PHAS*, only chr3-t, chr4-t/b and chr7-t in all five samples exhibited a biased distribution (Fig. 2 (blue arrow regions) and Fig. 4b). In chr4-t/b, the preference of 24-*PHAS* in chromosomes was very similar to that of 21-*PHAS*. In chr4-t, less *PHAS* loci were located in subA than in subB and subD (*P-value* < 1.0e-6), while in chr4-b, more *PHAS* loci were located in subA than in subB and subD (*P-value* < 2.2e-6). In chr3-t, more 24-*PHAS* were distributed in the subB genome than in the subA and subD genomes (*P-value* < 0.05). However, in chr7-t, far fewer 24-*PHAS* were located in the subB genome than in the subA and subD genomes (*P-value* < 1.0e-6). These data suggested that *PHAS* loci exhibited local chromosome preferences during the genome plasticity process.

### Homoeologous *PHAS* loci in the diploid, Tetraploid, and Hexaploid wheat

The progenitors of allohexaploid wheat contain the diploid genomes AA, BB and DD, and the tetraploid genome AABB. The genomes of AA (*Triticum urartu*, 2n = 2x = 14), DD (*Aegilops tauschii*, 2n = 2x = 14), and AABB (*Triticum turgidum*, 2n = 4x = 28) were sequenced, and their genome assembly was nearly perfect at the chromosome level. This made it possible to investigate the evolution of 21- and 24-*PHAS* in the different ploidy of *Triticum* species. Thus, we downloaded the genome sequences of AA, DD and AABB, and then mapped the 21- and 24-*PHAS* with scores of greater than 30, which were identified in these samples from the five developmental stages of inflorescence, to these genome sequences by the BLAST program with 80% identity and 80% matched sequence length. Approximately 22.91%~ 25.98% of the 21-*PHAS* could be mapped to the AA genome, and 37.77%~ 44.12% of the 21-*PHAS* could be mapped to the DD genome, which was slightly higher than the 21-*PHAS* that were mapped to the AA genome. However, a greater proportion of *PHAS* (60.83%~ 62.72%) could be mapped to the AABB genome. For 24-*PHAS*, the mapped proportions to AA, DD and AABB were very similar to the mapped 21-*PHAS*. Approximately 22.52%~ 26.13, 29.18%~ 37.42, and 53.96%~ 58.05% of 24-*PHAS* could be aligned to the AA, DD and AABB genomes, respectively (Supplementary Table 2). In the tetraploid AABB, the mapping rates of 21- and 24-*PHAS* were much higher than the diploid species AA and DD. The scatter diagram showed that the mapping rate was positively correlated with the ploidy times in 21-*PHAS* with r^2^ = 0.8019, as determined by Pearson correlation test (*P-value* = 6.41e-6, Supplementary Fig. 6A), and 24-*PHAS* with r^2^ = 0.8578 (*P-value* = 6.41e-6, Supplementary Fig. 6B). This indicated that the expansion of the whole genome led to an increase of the *PHAS* loci.

**Fig. 5:**
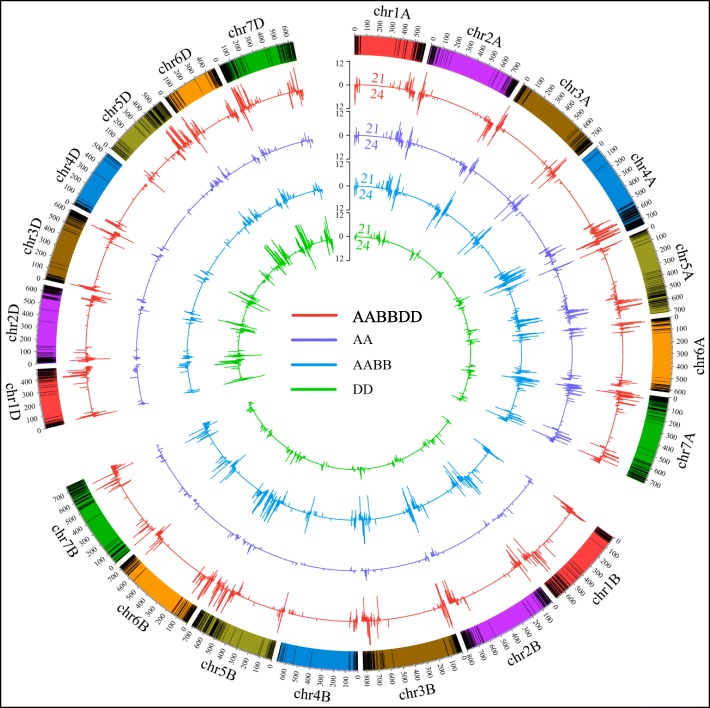
The density of 21- (top lines) and 24- (bottom lines) *PHAS* loci that can be mapped to the AABBDD (red lines), AA (purple lines), AABB (blue lines) and DD (green lines) genomes. The black bar represents the *PHAS* loci in each chromosome

For the mapped 21- and 24-*PHAS* to the AA genome, 67.55 and 75.73% of them were located in the subA genome of wheat, respectively. For the mapped 21- and 24-*PHAS* to DD genome, there were 72.78 and 77.53% of them located in subgenome D of wheat, respectively. For the mapped 21-*PHAS* to the AABB genome, 43.41 and 42.40% of them were from the subA and subB genome of hexaploid wheat, respectively, and for 24-*PHAS*, a similar proportion (i.e. 44.38 and 44.51% for subA and subB, respectively) was observed. The detailed genome distributions in the AABBDD genome for the mapped *PHAS* were clearly shown in each chromosome of Fig. 5. For the *PHAS* mapped to the AA genome, there were more peaks (purple lines in Fig. 5) from each chromosome of subA than from the other subgenomes. For the mapped *PHAS* to the DD genome, the peaks (green lines in Fig. 5) were mostly distributed in the subD genome. For the mapped *PHAS* to the AABB genome, each chromosome of both subA and subB had more peaks (blue lines in Fig. 5) than the subD genome. This finding indicated the orthologous relationship between each of subA and the AA genome, subB and BB genome, and subD and DD genome. The evolution independence of *PHAS* sequences among the three diploid species, AA, BB and DD, may suggest that the *PHAS* sequences in *Triticum* may diverge before tetraploid synthesis and may diverge after occurrence of the AA, BB and DD species.

### Genome distributions of MiR2118 and MiR2275 in *Triticum*

**Fig. 6:**
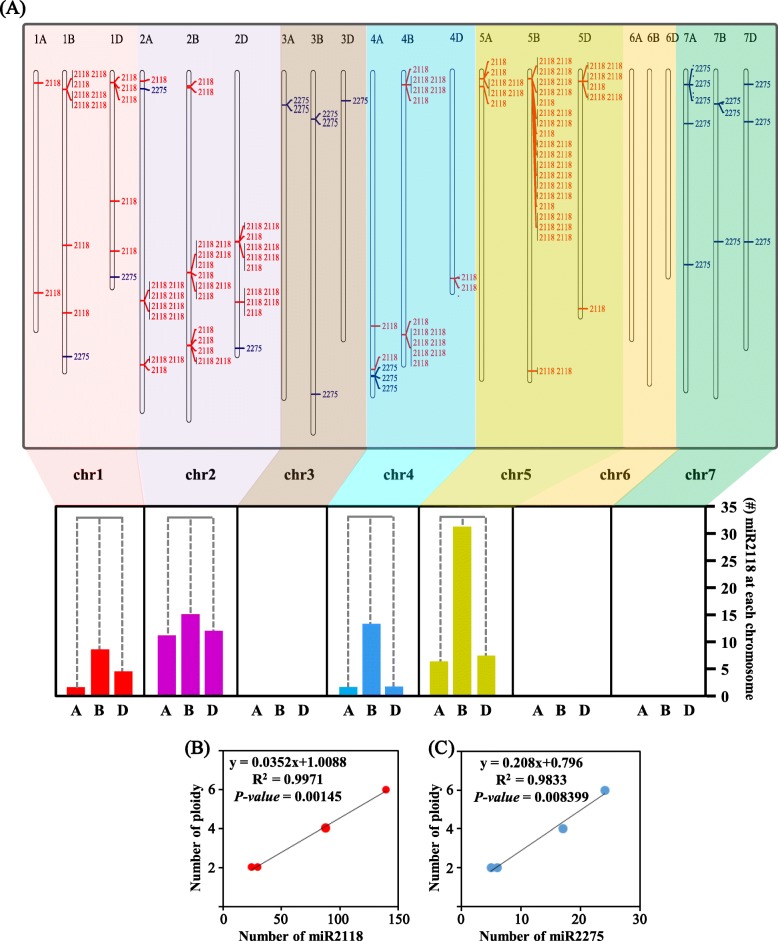
**a** The distribution of miR2118 (red lines) and miR2275 (blue lines) in each chromosome of wheat. **b-c** The correlation between the members of miR2118 **b** or miR2275 **c** and the times of ploidy. **a**, **b** and **d** represent the subA, subB and subD genomes, respectively

The production of phasiRNAs was initiated by miR2118 and miR2275 via cleavage of their *PHAS* precursors. MiR2118 and miR2275 possessed many copies in grasses. There were 18 members of miR2118 and four members of miR2275 in the rice genome and seven members of miR2118 and four members of miR2275 in the maize genome, based on the miRBase database [27]. To detect these miRNAs in *Triticum*, we mapped the mature sequences of miR2118 and miR2275 from the miRBase database to the *Triticum* genomes with perfect matches and found that there were 25, 30, 88, and 140 members of miR2118 in the DD, AA, AABB and AABBDD genomes, respectively, and 6, 5, 17 and 24 members of miR2275 in the four genomes, respectively. Most of these miRNAs were clustered on the chromosomes of the four species, as shown in Fig. 6a and Supplementary Fig. 7–8. The increase tendency of miR2118 and miR2275 was significantly correlated with ploidy, as determined by Pearson correlation test (*P-value* = 0.0015 and 0.0084, r^2^ = 0.9971 and 0.9833, respectively; Fig. 6b-c). For miR2118, there was also a biased distribution in the three subgenomes of wheat. However, unlike the 21-*PHAS*, the tendency of miR2118 on the subgenome in each group of chromosomes was consistent (Fig. 6a). Except for chr2, subB was significantly dominant, with many more members of miR2118 than the subA and subD genomes in chr1 (Fisher exactly test, *P-value* = 1.87e-5), chr4 (*P-value* = 3.90e-14) and chr5 (*P-value* = 3.65e-14). On chr2, there were also more members of miR2118 in the subB genome but not significantly with *P-value* > 0.05. In the tetraploid AABB genome (Supplementary Fig. 7), miR2118 in subB was also dominant than the subA genome on chr1 (*P-value* = 2.06e-8), chr2 (*P-value* = 1.43e-2), chr4 (*P-value* = 3.72e-10), and chr5 (*P-value* = 1.43e-2). The similar dominant subgenome distribution of miR2118 in the AABB and AABBDD genomes indicated that the dominance of subB may have occurred before the synthesized hexaploidy of wheat. In the AA and DD genomes, there were also fewer miR2118 in chr1, chr2, chr4 and chr5 than in subB of the AABB and AABBDD genomes (Supplementary Fig. 8A-B), which indicated that the expansion of miR2118 in the subB genome may have occurred before the synthesized tetraploidy of the AABB genome. This indicated the dynamic expansion of the trigger miRNAs following genome expansion or polyploidization.

### *PHAS* loci as the targets of MiR2118 and MiR2275 in wheat

The production of the *PHAS* loci could be initiated by miR2118 and miR2275, and then generate the 21- and 24-nt phasiRNAs, respectively. To identify whether miR2118 and miR2275 also target these *PHAS* transcripts in wheat, using the Targetfinder program [28], we aligned the two miRNA families to the *PHAS* sequences with a score less than 4. Taking these samples of the five development stages as examples, 35.43% (767 out of 2159), 34.06% (220 out of 646), 35.31% (446 out of 1263), 38.31% (824 out of 2151), and 35.09% (1575 out of 4488) of 21-*PHAS* sequences were predicted to be targeted by miR2118 in AM, ST, FHM, MIT, and MP, respectively. For 24-*PHAS*, 43.36% (575 out of 1323), 50.43% (523 out of 1037), 50.26% (591 out of 1176), 0.45% (9 out of 1991), and 48.86% (1111 out of 2274) were identified as the targets of miR2275, respectively. The alignment information between the miRNAs and *PHAS* loci were listed in Supplementary Table 3, 4, 5, 6, 7.

**Fig. 7:**
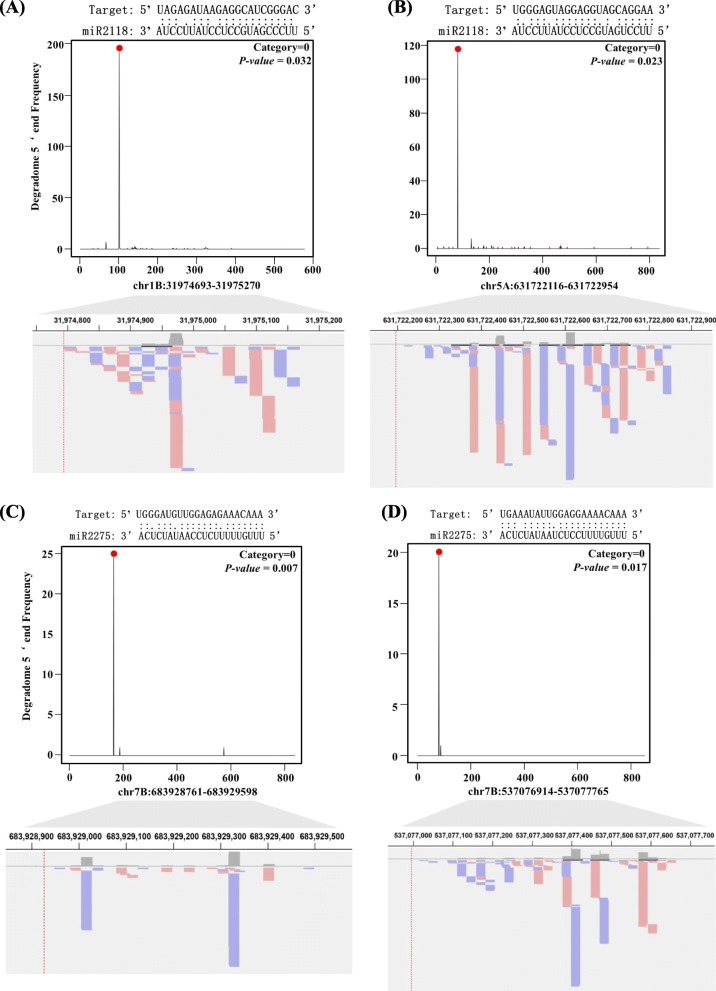
Target plots (T-plots) of miRNAs characterized in the degradome datasets. The abundance of signature tags was plotted along the indicated transcripts. The red dots indicate the predicted cleavage sites on the x-axis, and the black lines indicate the signatures produced by miRNA-directed cleavage. In the bottom panel, the red or blue lines represents the positive or negative strand orientation of the phasiRNAs

To validate whether these *PHAS* transcripts can be indeed cleaved by miR2118 and miR2275, we downloaded the degradome sequences from the GEO datasets for the young spike tissue under cold stress by Song et al [29], which corresponds to the small RNA datasets of the AM stage of young spike (control samples, SRR3680677 and SRR3680678; and cold stress samples at 0 °C after 48 h, SRR3680679 and SRR3690680). According to the abundance of reads along the whole transcripts, using the Cleaveland program [30] (*P-value* < 0.05 and the category <= 2), miR2118 and miR2275 were considered to be interacted between *PHAS* transcripts and miRNAs. The target plots of miR2118 and miR2275 characterized in the degradome datasets were shown in Fig. 7a-d. And at the cleavage sites, the generated phasiRNAs with orientation from the positive or negative strand were shown in the bottom panel of Fig. 7a-d. A total of 13.01% (520 out of 3996, SRR3680679) and 12.80% (525 out of 3996 in SRR3680680) of 21-*PHAS* were confirmed to be cleaved by miR2118 for the cold stressed samples. In the control samples, slightly less 21-*PHAS* were validated as cleaved targets, i.e. 9.86% (355 out of 3601 in SRR3680677) and 9.65% (353 out of 3659 in SRR3680678). For miR2275, few 24-*PHAS* were detected to be cleaved in the control and cold stressed samples. Only 0.18% (two out of 1122 in SRR3690680) and 0.38% (four out of 1052 in SRR3680679) of the 24-*PHAS* in the cold stressed samples were validated to be cleaved by miR2275, while in the control samples, there were slightly more target sites of 24-*PHAS* than in the cold stress samples, as confirmed by cleavage of miR2275, i.e. 3.16% (45 out of 1421 in SRR3680677) and 9.65% (38 out of 1203 in SRR3680678). This finding provided evidences that miR2118 and miR2275 could mediate the cleavage of *PHAS* transcripts in wheat. The cleavage information of the degradome for the *PHAS* loci were listed in Supplementary Table 8, 9, 10.

### The expression level of *PHAS* loci

**Fig. 8:**
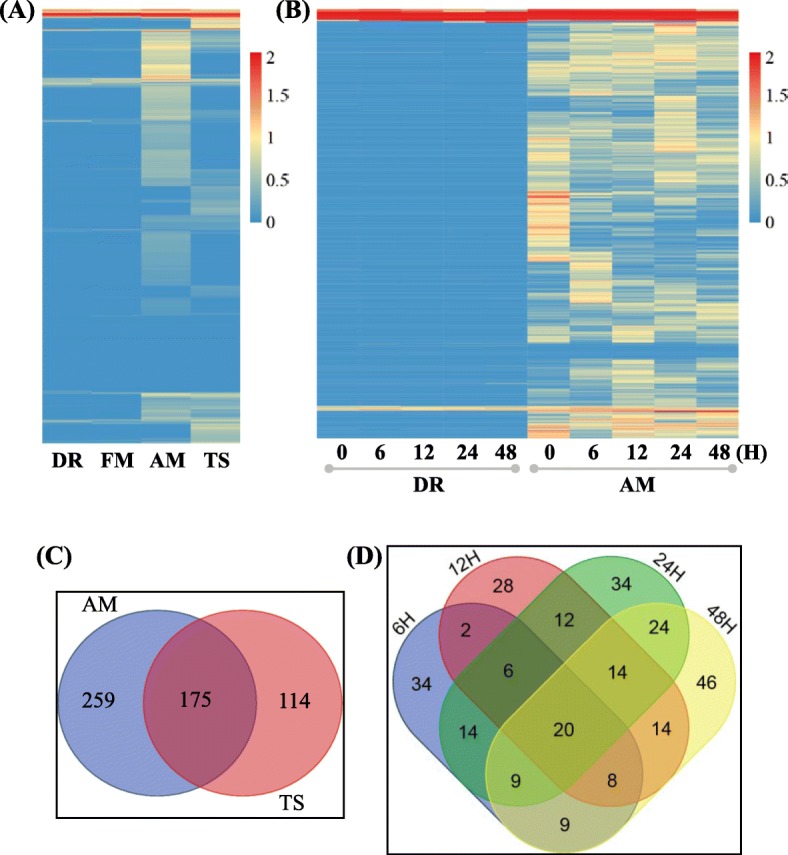
Expression heatmap of *PHAS* transcripts in the young spike of DR, FM, AM and TS, and in the cold-stressed young spike transcriptome with polyA after 0, 6, 12, 24 and 48 h in DR and AM

The generation of phasiRNAs may depend on the expression of their precursors. To detect the expression of the *PHAS* precursors, we downloaded the RNA-seq datasets from the GEO database, including the reproductive tissues at DR, FM, AM and TS. The *PHAS* precursors were mostly expressed in the AM and TS stages, and only a few of them were expressed in the DR and FM stages (Fig. 8a). It was coordinated with the expression of phasiRNAs. Furthermore, most of their expression levels were very low. Only 434 and 289 of *PHAS* with the expression level were more than one RPKM in the AM and TS stages of young spikes, respectively, and 175 of them were overlapped between the two stages (Fig. 8c). This finding indicated the specific expression of *PHAS* loci in different developmental stages of young spikes.

*PHAS* transcripts were expressed specifically in the reproductive tissues, but whether they responded to abiotic stress was still unknown until now. Taking cold stress as an example, using the transcriptomes with polyA in the DR and AM stages of young spike samples including 0 (control), 6, 12, 24 and 48 h (hours) after cold stress at 0 °C, we identified the expression of these *PHAS* loci. In the cold stressed samples, most of the *PHAS* loci were also mainly expressed in the AM stage, and the expression level also showed a difference (Fig. 8b). Using the DEseq program [31] with the fold change (stressed vs control) more than two times and adjusting the *P-value* less than 0.01, 102, 104, 133 and 144 of *PHAS* were characterized as differentially expressed *PHAS* transcripts at 6, 12, 24 and 48 h after cold stress at 0 °C, respectively. For the differentially expressed *PHAS*, a total of 66.67% (68 out of 102), 73.08% (76 out of 104), 74.43% (99 out of 133) and 68.06% (98 out of 144) were common at the four stressed time points (Fig. 8d). These results indicated that the *PHAS* transcripts responded to abiotic stress, such as cold stress.

## Discussion

### High conservation of the biogenesis mechanism and low conservation of *PHAS* sequences in plants

PhasiRNAs are present in many grass family plants including rice [3, 9, 16, 17], maize [8], *Brachypodium* [32]*, sorghum* [1], *foxtail millet* [1], and litchi [15]. They play important roles during anther development [8]. Recent studies showed that the biogenesis mechanisms of phasiRNAs and their functional modes on target genes are conserved among grasses plants [8, 9, 15–17]. Despite this, the *PHAS* transcripts have very high diversity in the sequence types in different grass species. By comparing these *PHAS* loci in wheat to the genomes of their closely related species, including *Brachypodium*, rice, sorghum and maize, only 17, 7, 7 and 5 *PHAS* sequences in wheat could be matched with the 50% identity and 50% matched length, respectively. This result indicated that *PHAS* is less conserved across grass species. *PMST1* producing 21-*PHAS* triggered by miR2118, as a typical example, regulates photoperiod-sensitive male sterility in rice [23], whereas *PMST1* exists only in rice without a homolog in other grasses. Thus, *PHAS* and phasiRNAs are highly species-specific in grasses.

There is a special class of *PHAS*, i.e. *TAS* genes. Until now, ten *TAS* genes were characterized in plants. For *TAS* genes, only some of them have high conservation, and most of them are species-specific and involved in reproductive process and biotic or abiotic stresses [2, 33, 34]. *TAS1* and *TAS2*, which are initiated by miR173, are conserved in eudicots [33, 34]. *TAS3*, which is triggered by miR390, is conserved in land plants [35]. Some of the *TAS* genes are family-specific. *TAS5*, *TAS9* and *TAS10* are Solanaceae-specific [36, 37]. The other *TAS* genes are species-specific. The *TAS4* gene only exists in *Arabidopsis* [38]. *TAS6* only exists in moss [39], and *TAS7–10* only exists in grapevine [40]. These *TAS* genes are involved in various biological processes, such as Auxin signaling, heat and chilling response [2].

Another category of *PHAS* is involved in regulating plant natural immunity. The regulation mechanism is also conserved both in eudicots and monocots. However, the trigger miRNAs are not conserved between eudicots and monocots. In eudicots and gymnosperm, miR472, miR482/2118, mir6024, and miR1507 can target *NB-LRR* transcripts and initiate the generation of phasiRNAs [41]. Most of these *NB-LRR* genes contain the *TIR* domains [42]. In monocots, very few miRNAs were identified to target the *NB-LRR* genes [41]. Recently, in *Triticum*, several miRNA families, such as miR9863, miR3117, miR3084, miR5071 and miR7757, were characterized to target *NB-LRR* transcripts and trigger the production of phasiRNAs in wheat [41]. And most of them are absent or lost function in other grass species. In *Triticum*, there is also variation in the expression level or copy number for the five miRNAs [41]. The relationship between miRNAs and *PHAS* may frequently become absent and present during the evolution of plants.

There are no common miRNAs in eudicots or monocots that regulate the *NB-LRR* genes. Intriguingly, miR2118, a common miRNA in eudicots and monocots diverged to neofunctionalization. In eudicots, miR2118/482 was found to mostly target *NB-LRR* genes and initiate the generation of 21-nt phasiRNAs [13]. In contrast, miR2118 in monocots mostly targets numerous noncoding sequences and triggers the generation of 21-nt phasiRNAs [3, 8, 16]. MiR2118 in eudicots is involved in plant immunity response [13], while in monocots, miR2118 plays an important role in anther development [8, 9]. In addition, miR2118 in grasses such as rice [3] and wheat (Fig. 6) undergoes a special tandem repeat expansion. The function variation of miR2118 in the plant evolution process may be due to the target sequence variation. The sequences of the *NB-LRR* genes rapidly diversified between eudicots and monocots [42]. For example, the *TIR* domain is present in most eudicots but absent in monocots. The domain loss or sequence variation of the *NB-LRR* genes may lead to the functional variation of their regulator.

### Dynamic evolution of *PHAS* loci and their trigger MiRNAs in *Triticum*

**Fig. 9:**
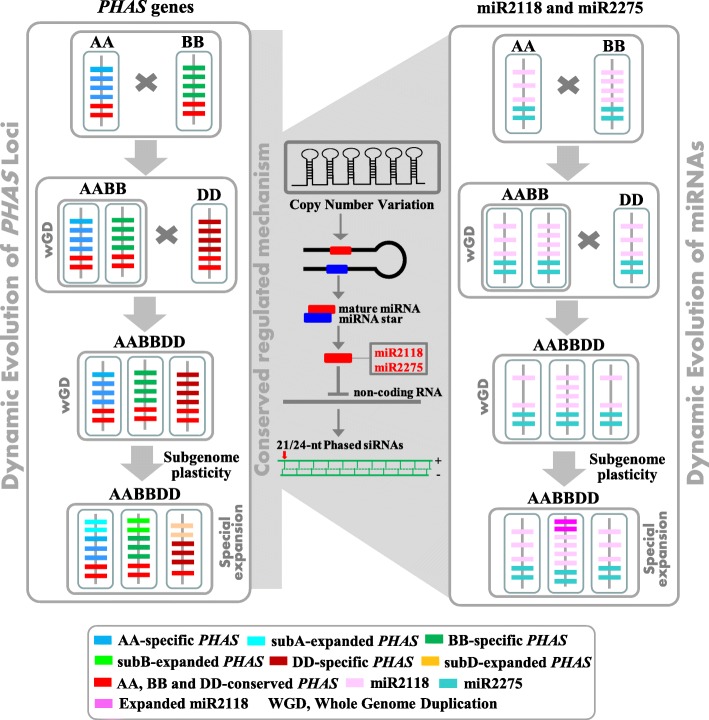
Dynamically evolved *PHAS* (the left panel) and their regulators miR2118 and miR2275 (the right panel) in the diploid, tetraploid and hexaploid *Triticum* species

In grasses, numerous *PHAS* loci have been identified in maize [8], rice [3, 9] and wheat. Most of them derived from the non-coding regions or intergenic regions (Supplementary Fig. 3). These *PHAS* loci have very high species specificity, as described in section 3.1. The *Triticum* species had a small time-scale in the divergence history. In *Triticum*, the progenitors of wheat AA, and DD diverged approximately four million years ago (mya). AABB and AA diverged approximately 0.5 mya, and AABBDD were nascent approximately 10,000 years ago. In the small time-scale of evolution, the *PHAS* loci still have high divergence. Most of the *PHAS* loci (76.09~86.22%) were singletons with only one homoeolog among the three subgenomes, and others possessed triplet and duplet homoeologs in wheat (Supplementary Fig. 5). This study demonstrated the lower conservation of *PHAS* among the three subgenomes. Approximately 22–25%, 61–62% and 37–44% of *PHAS* in AABBDD could be mapped to the AA, AABB and DD genomes, respectively. The lower identity of *PHAS* loci among subA, subB and subD indicated high heterogenization among the three subgenomes. Intriguingly, most of the *PHAS* mapped to AA, AABB and DD were located in the subA, subA & B and subD genomes of wheat, respectively. This indicated that the variation of *PHAS* among subA, subB and subD may be after the divergence of the AA, BB and DD species and may occur before the synthesized AABB and AABBDD (the left panel of Fig. 9). The subgenome plasticity may contribute less to the divergence of *PHAS* among the three subgenomes (the left panel of Fig. 9). The *PHAS* that were not mapped to the AA, AABB or DD genomes may be the nascent *PHAS* after the yield of wheat. Overall, the non-coding *PHAS* loci may have a much higher evolution rate than the protein-coding genes.

The trigger miRNAs of *PHAS* were also expanded following genome expansion or polyploidization. The members of miR2118 and miR2275 in diploid, tetraploid and hexaploid were progressively increased and positively related to the ploidy. This result indicated the lower subgenome plasticity of the miRNA trigger among the three sets of genomes in wheat. More miR2118 was distributed in most subB chromosomes in AABB and AABBDD than in subA and subD (Fig. 6a and Supplementary Fig. 7). However, there was no evidence to deduce the occurrence times of miR2118 expansion in the subB genome. Nevertheless, in chr5B of the AABBDD genome, there were 35 members of miR2118 (Fig. 6a), which was more than the 17 members of miR2118 in chr5B of the AABB genome (Supplementary Fig. 7). This indicated the special expansion of miR2118 in chr5B of AABBDD genomes, and the expansion may occur after the synthesis of hexaploid wheat (right panel of Fig. 9).

The expansion of miR2118 in *Triticum* and the other grass species may be associated with the numerous *PHAS*. Increasing the number of copies of miR2118 may enhance the ability to regulate *PHAS* and yield phasiRNAs (the middle panel of Fig. 9). After initiating the cleavage of the mRNA precursors of phasiRNAs, the secondary 21-nt phasiRNAs can function in *cis* to target their own precursors, which was observed in both rice and maize [9]. The simultaneously expanding and subgenome plasticity in both miRNAs and *PHAS* of the polyploid *Triticum* species may indicate their co-evolution.

### *PHAS* loci involved in male sterility

Numerous *PHAS* loci were observed in male reproductive organs, such as young spikes and anthers in grasses. The *PHAS* loci were closely related to the fertility of the anther. Direct evidence is that the presence of *PMST1* showed that *PHAS* was involved in male sterility in rice [23]. Indirect evidence is the mutants of genes in the biogenesis pathway of phasiRNAs. The *DCL4* mutants in rice resulted in severe developmental defects in the spikelet, such as slight opening between the lemma and the palea. The lemma was partially or completely degenerated to the awn, and the strong loss-of-function transgenic plants were sterile [43]. A marked reduction of 21-nt phasiRNAs was observed in the *osdcl4–1* panicles [16]. The *OsDCL3b* RNAi lines led to reduced pollen fertility, seed setting rate and decreased grain yield in rice [44]. In addition, *osdcl3b* mutants specifically affected the generation of 24-nt phasiRNAs [16]. The *osrdr6–1* mutant was temperature sensitive and exhibited spikelet defects. The *osrdr6–1* mutants had a strong impact on the accumulation of both 21- and 24-nt phasiRNAs [17]. MEIOSIS ARRESTED AT LEPTOTENE1 (MEL1), a rice AGO, was shown to function in the development of pre-meiotic and meiotic procession, which preferred to load 24-nt phasiRNAs that bear a 5′-terminal cytosine [18]. In addition, *mel1* mutants also behaved sterile phenotype [45]. In the micorspore mother cell (MMC) stage and meiotic prophase (MP) stage of male sterile wheat, the miR2275-3p was the significantly down-regulated compared to the fertility lines and the 24-nt siRNAs also had lower abundance in male sterile wheat lines [46]. In our study, the 24-nt phasiRNAs and miR2275 were abundant simultaneously in the TS stage (Fig. 1c-d), and validated by the degradome datasets to recognize the target sites (Fig. 7c-d & Supplementary Tables 9, 10, 11) and mediate the generation of phasiRNAs. Therefore, miR2275 and 24-nt phasiRNAs may be also associated with the male sterility in wheat. The multiple copy number of miR2275 in the genome of wheat (Fig. 6a) also indicated the importance of phasiRNAs in the evolution of plants. However, how *PHAS* leads to male sterility remains unclear. Whether all of the *PHAS* loci or phasiRNAs are associated with anther development or whether only several key *PHAS* loci or phasiRNAs play important roles is also unclear. The resolution of mysteries of *PHAS* in anther development remains a challenge for future studies.

### *PHAS* or phasiRNAs may be responsive to abiotic stresses

The *PHAS* loci were preferentially identified in reproductive organs in grass species, which indicated their expression preference. Similar to phasiRNAs, few *PHAS* transcripts were expressed in the DR and FM of young spikes (Fig. 8a & b), which were also found in maize [8] and rice [9]. Most of the *PHAS* in reproductive organs in wheat had low expression levels, and only 10.49 and 4.27% of the *PHAS* in the AM and TS stages had expression levels with RPKM > = 1 (Fig. 8c). The expression level between AM and TS was also different (Fig. 8c). In addition, 274 *PHAS* in AM were differentially expressed at 6, 12, 24, and 48 h after cold stress at 0 °C (Fig. 8d). This result indicated that *PHAS* not only involved in anther development but also could respond to cold stress. In addition to cold stress, the *PHAS* may also be sensitive to high temperatures in the development of anthers or flowers. The phenotype of *osrdr6* mutants was temperature dependent in rice. The phenotypic severity of *osrdr6–1* was enhanced with increased growth temperature under the fixed photoperiod. At lower temperatures of 28 °C or 30 °C, no obvious developmental defect or only 10% of the mutant spikelets showing a slight opening between lemma and palea was observed. At higher temperatures, the phenotypes of the mutant spikelets were very severe. At 32 °C, the lemma was lost or became a completely or partially radial, awn-like structure or both the lemma and the pelea degenerated into awn-like structures. At 34 °C, most of the lemma and pelea were defective, with shriveled or filament-like anthers. Both 21- and 24-nt phasiRNAs decreased in *osrdr6* at higher temperatures [17]. Thus, phasiRNAs or *PHAS* transcripts may be temperature dependent or thermo-sensitive. In addition to the thermo-response, some *PHAS* were photoperiod-sensitive, which affected the transition of male fertility and sterility under different photoperiod conditions. 337S is a male sterile wheat line that is sensitive to both long day-length/high temperature and short day-length/low temperature condition. In 337S male sterile line, the miR2275 was down-regulated in MMC and MP stage [46]. MiR2275 could mediate the generation of 24-nt phasiRNAs. Thus, temperature and photoperiod conditions may affect the generation of phasiRNAs in wheat. Under long-day conditions, *PMS1T* was targeted by miR2118 to produce 21-nt phasiRNAs, which affected rice fertility. Under short-day conditions or under long-day conditions with the mutated target site of miR2118, *PMS1T* could not produce 21-nt phasiRNAs because the transcript of *PMS1T* could not be recognized by miR2118 [47]. Overall, according to these features of *PHAS and* their associated key genes, the photothermo-sensitive genic male sterile lines may be a good model to study the further function of phasiRNAs or *PHAS*, and the detailed molecular mechanism of male sterility will be uncovered.

## Methods

### Identification of PhasiRNAs and *PHAS* loci

The small RNA datasets used in this study were downloaded from the NCBI GEO database (Supplementary Table 1). The reference genomes of AA (*Triticum urartu* (v1), 2n = 2x = 14), DD (*Aegilops tauschii* (v1), 2n = 2x = 14), AABB (*Triticum turgidum* (v1), 2n = 4x = 28) and AABBDD (*Triticum aestivum* (refv1.0), 2n = 6x = 42) were obtained from the URGI website (https://wheat-urgi.versailles.inra.fr/Seq-Repository/Assemblies). Next, the Trimmomatic program (version 0.38) was used to screen the raw small RNA datasets and remove the adaptor sequences and contaminated reads. Subsequently, the ShortStack program (version 3.8.5) [19] was used to align the cleaned data to the wheat reference genome (AABBDD). Then, the distribution of small RNAs on the reference genome was analyzed by the perl scripts, and *PHAS* loci were identified with phased scores of greater than 15, 20, 25 or 30. According to the genome location of *PHAS*, the *PHAS* loci were annotated. A detailed flowchart was shown in Supplementary Fig. 1.

### The genome distribution of *PHAS* loci and their trigger MiR2118 and MiR2275

To show the distribution of *PHAS* loci on the wheat genome, the total number of *PHAS* loci in each 500 kb window sliding of each chromosome was calculated. Then, we used the Circos program (version 0.69–6) [48] to show the number of 21-*PHAS* (red lines in Figs. 2, 3 and 5) and 24-*PHAS* (blue lines in Figs. 2, 3 and 5) loci in each chromosome in the wheat genome. The homoeologous chromosomes were filled with the same colors as the rainbow chromosomes (Figs. 2, 3 and 5). The black lines in the rainbow chromosomes indicated the *PHAS* loci. The homoeologous *PHAS* loci among the homoeologous chromosomes were linked with the same color lines, and others were linked with different color lines (Fig. 3).

To identify miR2118 and miR2275, the mature sequences of the two families of miRNAs were downloaded from the miRBase website (http://www.mirbase.org/). Then, we mapped these sequences to the AA, DD, AABB, and AABBDD genomes using the BLAST program [49] with perfect matches. The distributions of miR2118 and miR2275 on each chromosome were drawn with the Mapchart program of the R package [50].

### Target analysis of *PHAS* transcripts

To understand whether the *PHAS* loci were regulated by miR2118 and miR2275 in wheat, we used the Targetfinder program [28] to predict the miRNA target sites in the *PHAS* sequences with scores less than four. To validate whether the miRNAs can indeed cleave the predicted targets, we downloaded the degradome (SRP076763) datasets from the GEO database, including the control and cold treatment datasets [29]. The degradome datasets with the control and cold stress samples corresponded to the small RNA datasets for the control (SRR3680677 and SRR3680678) and cold stress samples (SRR3680679 and SRR3690680). Then, we used the two degradome libraries to confirm the target cleavage sites in the identified *PHAS* sequences in their corresponding control and cold stressed small RNA datasets with the CleaveLand program [30]. The cleavage sites were classified into 0, 1, 2, 3, and 4 categories according to the abundance of reads in the cleaved sites along the whole transcripts with a *P-value* less than 0.05. At the cleaved sites, the categories represented the following categories: category 4, only one read located at that position; category 3, > 1 read but below or equal to the average depth of coverage on the transcript; category 2, > 1 read above the average depth but not the maximum on the transcript; category 1, > 1 read, equal to the maximum on the transcript when there was > 1 position at the maximum value; and category 0, > 1 read equal to the maximum on the transcript when there was just 1 position at the maximum value. Here, only these cleavages with the category <= 2 and *P-value* < = 0.05 were selected for further study.

### The expression level of *PHAS* transcripts

To understand the expression level of *PHAS* transcripts, we downloaded the transcriptome data of DR (SRR5464507 and SRR5464508), FM (SRR5464515 and SRR5464518), AM (SRR5464519 and SRR5464520) and TS (SRR5464523 and SRR5464524) stages of young spikes, which corresponded to the following small RNA datasets: DR (SRR5460930 and SRR5460939), FM (SRR5460941 and SRR5460949), AM (SRR5460967 and SRR5460972) and TS (SRR5461176 and SRR5461177), respectively. Then, the RSEM program [51] was used to calculate the expression value (RPKM) of the *PHAS* transcripts. To investigate whether these *PHAS* loci responded to abiotic stress, we compared the transcriptomes of control and cold stressed samples in the DR and AM of young spikes. Then, we used the DEseq program [31] to calculate the differentially expressed transcripts. The heatmaps of the *PHAS* transcripts were drawn using the Pheatmap program in the R package.

## Conclusions

In summary, our results provided the first *PHAS* profiles in the young spike of wheat. They were also triggered by miR2118 and miR2275 to generate the phasiRNAs, which provided another evidence for the conservation of *PHAS* loci in generation and regulation mechanism of grasses. The increase of genome ploidy was the major drive force for the expansion of *PHAS* loci and their trigger miRNAs in *Triticum*. The sequence variations and biased distribution of *PHAS* in these genomes of AA, DD, AABB and AABBDD suggested their origination and diversity with rapid evolution in a small time-scale. Further study of the molecular mechanism for these *PHAS* loci may improve our understanding of the function of phasiRNAs in the male reproductive developments of modern polyploid wheat.

## Supplementary information

**Additional file 1: Supplementary Figure 1.** The flowchart of the identification of *PHAS* loci.

**Additional file 2: Supplementary Figure 2.** The number of *PHAS* loci in different tissues, including vegetative tissues such as leaves and reproductive tissues such as grain, spikelet, seeds, young spikes and anthers.

**Additional file 3: Supplementary Figure 3.** The proportions of 21- and 24-*PHAS* located in gene regions, repeat sequence regions and intergenic regions.

**Additional file 4: Supplementary Figure 4.** Venn diagram of 21- (A) and 24-*PHAS* loci (B) in AM, TS, FHM, MIT and MP.

**Additional file 5: Supplementary Figure 5.** Distribution of *PHAS* copies in the AM, TS, FHM, MIT and MP stages of hexaploid wheat.

**Additional file 6: Supplementary Figure 6.** The correlation between the percentage of mapped 21- and 24-*PHAS* to the AA, DD and AABB genomes and the times of ploidy.

**Additional file 7: Supplementary Figure 7.** The distribution of miR2118 (red lines) and miR2275 (blue lines) in each chromosome of the AABB genome.

**Additional file 8: Supplementary Figure 8.** The distribution of miR2118 (red lines) and miR2275 (blue lines) in the AA (**A**) and DD (**B**) genomes.

**Additional file 9: Supplementary Table 1.** Identified 21- and 24-*PHAS* with phased scores greater than 15, 20, 25 and 30 in 261 small RNA samples.

**Additional file 10: Supplementary Table 2.** The number and proportion of mapped 21- and 24-*PHAS* with scores greater than 30 for the AA, AABB and DD genomes.

**Additional file 11: Supplementary Table 3.** The predicted targets in AM with scores less than four, as determined by the Targetfinder program.

**Additional file 12: Supplementary Table 4.** The predicted targets in TS with scores less than four, as determined by the Targetfinder program.

**Additional file 13: Supplementary Table 5.** The predicted targets in FHM with scores less than four, as determined by the Targetfinder program.

**Additional file 14: Supplementary Table 6.** The predicted targets in MIT with scores less than four, as determined by the Targetfinder program.

**Additional file 15: Supplementary Table 7.** The predicted targets in MP with scores less than four, as determined by the Targetfinder program.

**Additional file 16: Supplementary Table 8.** The target cleavage information in AM (control: SRR3690677) from the degradome datasets.

**Additional file 17: Supplementary Table 9.** The target cleavage information in AM (control: SRR3690678) from the degradome datasets.

**Additional file 18: Supplementary Table 10.** The target cleavage information in AM (cold stress: SRR3690679) from the degradome datasets.

**Additional file 19: Supplementary Table 11.** The target cleavage information in AM (cold stress: SRR3690680) from the degradome datasets.

## Data Availability

These small RNA, transcriptome, and degradome datasets were downloaded from the NCBI GEO and SRA database. The datasets supporting the conclusions of this article are included within the article and its additional files.

## Abbreviations

AM: Anther primordia stage
DR: Double-ridge stage
FHM: Free haploid microspores stage
FM: The stage of appearance of the floret meristems
MIT: Mitosis stage
MP: Mature pollen
NT: Nucleotide
*PHAS*: PhasiRNA precursors
PhasiRNAs: Phased, secondary small interfering RNAs
TPM: Transcripts Per Million
TS: Tetrads stage
