# Supplementary figures and images for: Evolution of PHAS loci in the young spike of Allohexaploid wheat
Source: BMC Genomics. 2020 Mar 4;21:200. doi: 10.1186/s12864-020-6582-4 (PMC7057497; doi:10.1186/s12864-020-6582-4)

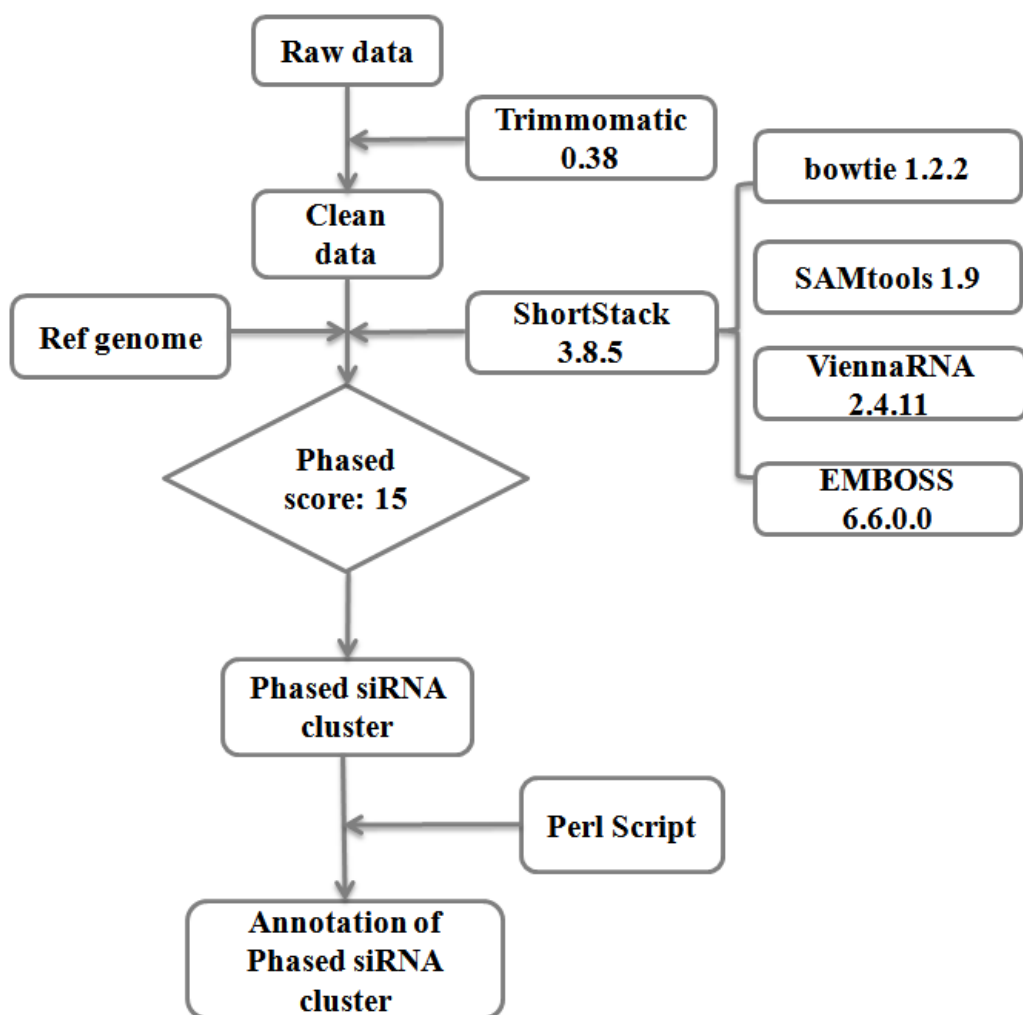

Supplement: Supplementary file 1 — Additional file 1: Supplementary Figure 1. The flowchart of the identification of PHAS loci. [file 12864_2020_6582_MOESM1_ESM.pdf]

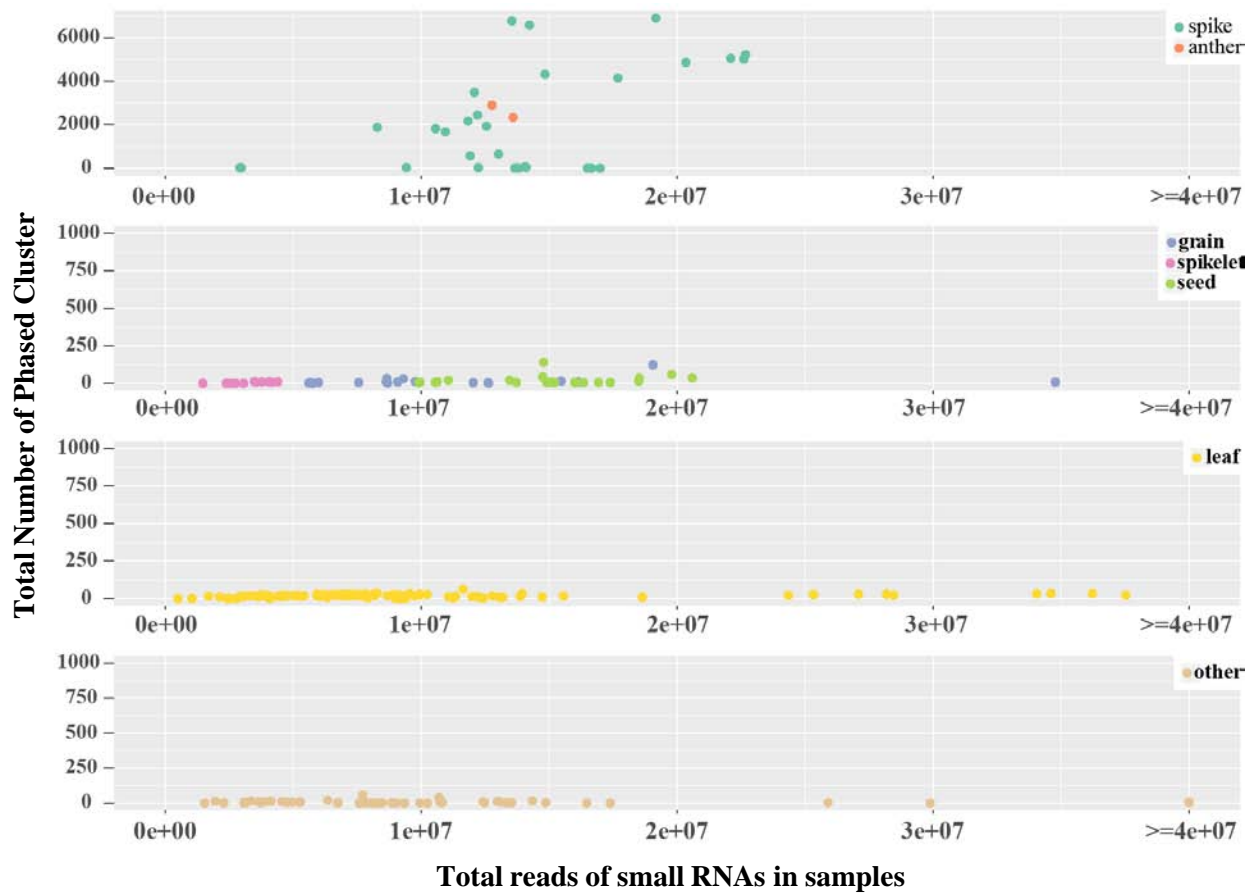

Supplement: Supplementary file 2 — Additional file 2: Supplementary Figure 2. The number of PHAS loci in different tissues, including vegetative tissues such as leaves and reproductive tissues such as grain, spikelet, seeds, young spikes and anthers. [file 12864_2020_6582_MOESM2_ESM.pdf]

(A)

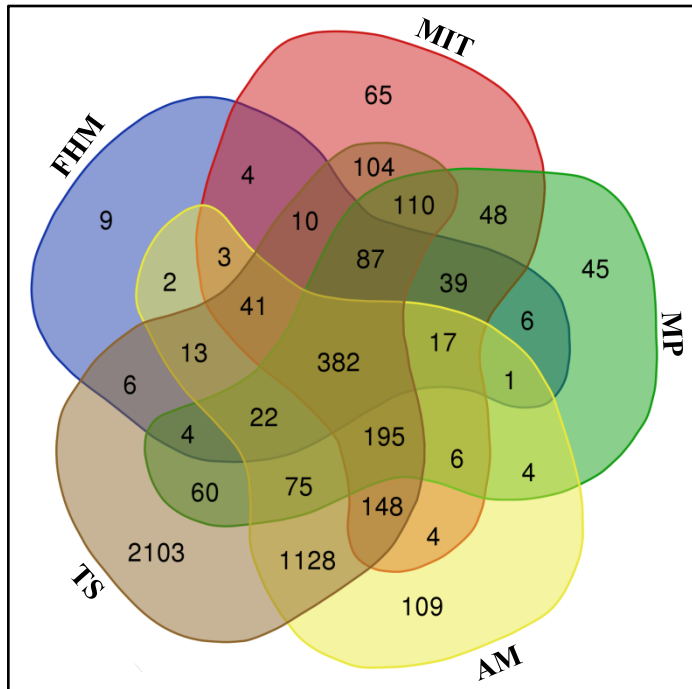

(B)

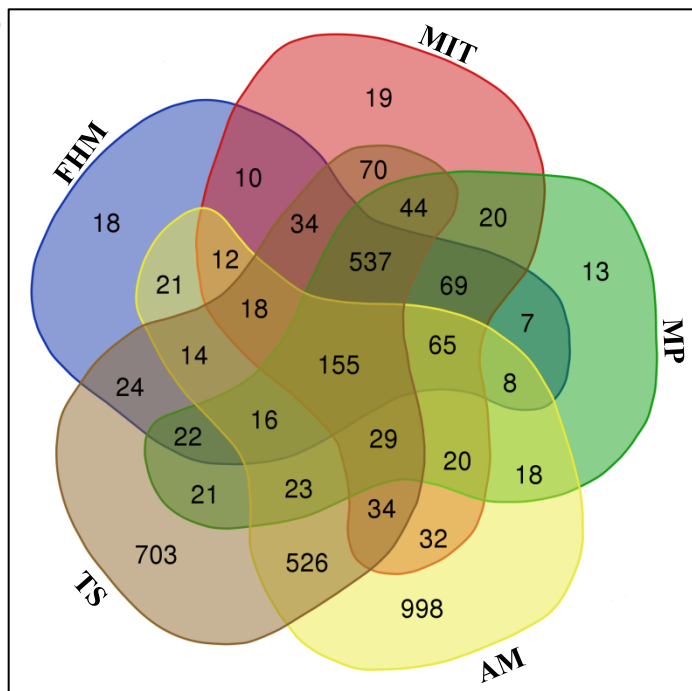

Supplement: Supplementary file 4 — Additional file 4: Supplementary Figure 4. Venn diagram of 21- (A) and 24-PHAS loci (B) in AM, TS, FHM, MIT and MP. [file 12864_2020_6582_MOESM4_ESM.pdf]

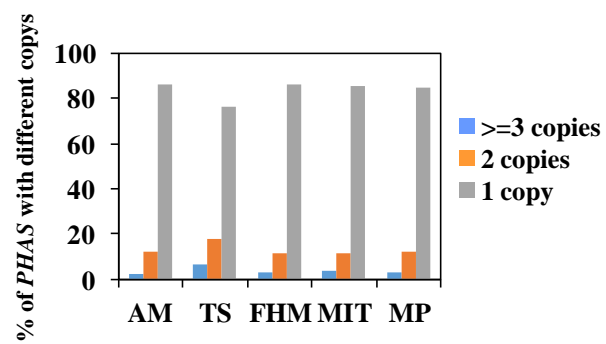

Supplement: Supplementary file 5 — Additional file 5: Supplementary Figure 5. Distribution of PHAS copies in the AM, TS, FHM, MIT and MP stages of hexaploid wheat. [file 12864_2020_6582_MOESM5_ESM.pdf]

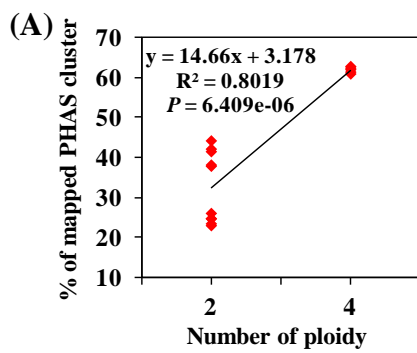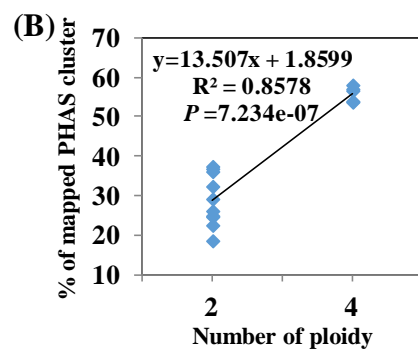

Supplement: Supplementary file 6 — Additional file 6: Supplementary Figure 6. The correlation between the percentage of mapped 21- and 24-PHAS to the AA, DD and AABB genomes and the times of ploidy. [file 12864_2020_6582_MOESM6_ESM.pdf]

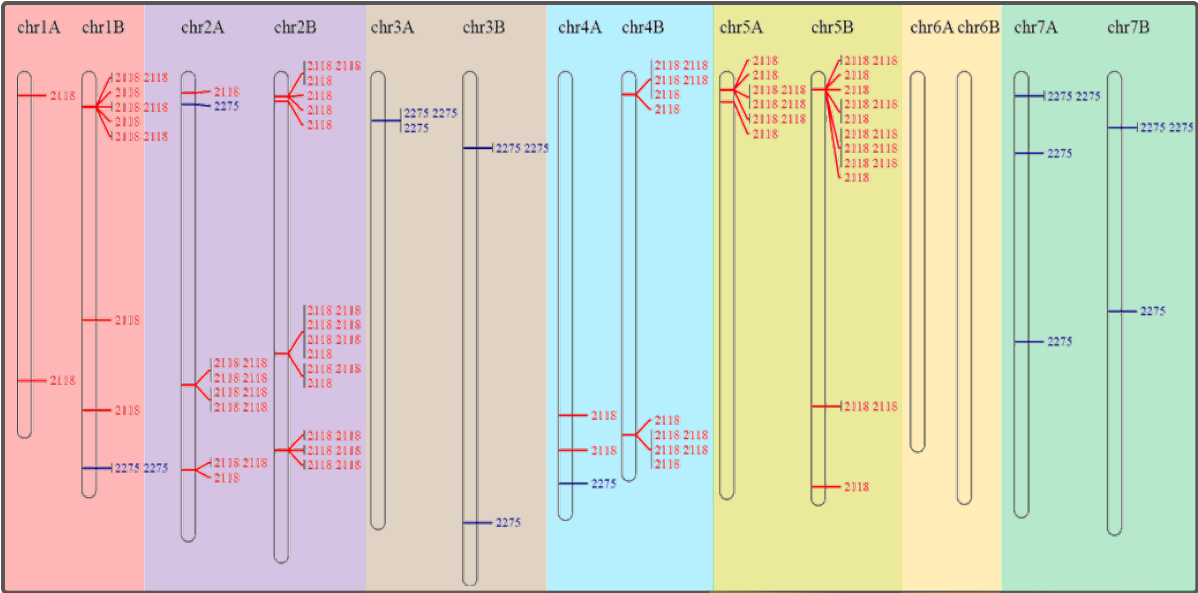

chr1

chr2

chr3

chr4

chr5

chr6

chr7

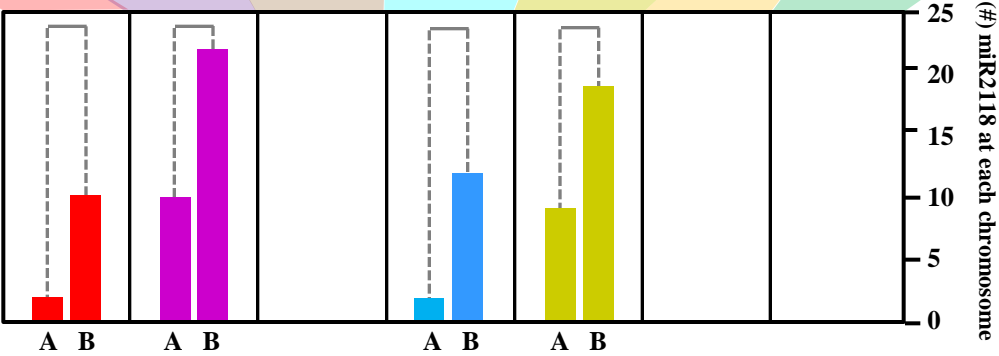

Supplement: Supplementary file 7 — Additional file 7: Supplementary Figure 7. The distribution of miR2118 (red lines) and miR2275 (blue lines) in each chromosome of the AABB genome. [file 12864_2020_6582_MOESM7_ESM.pdf]

(A)

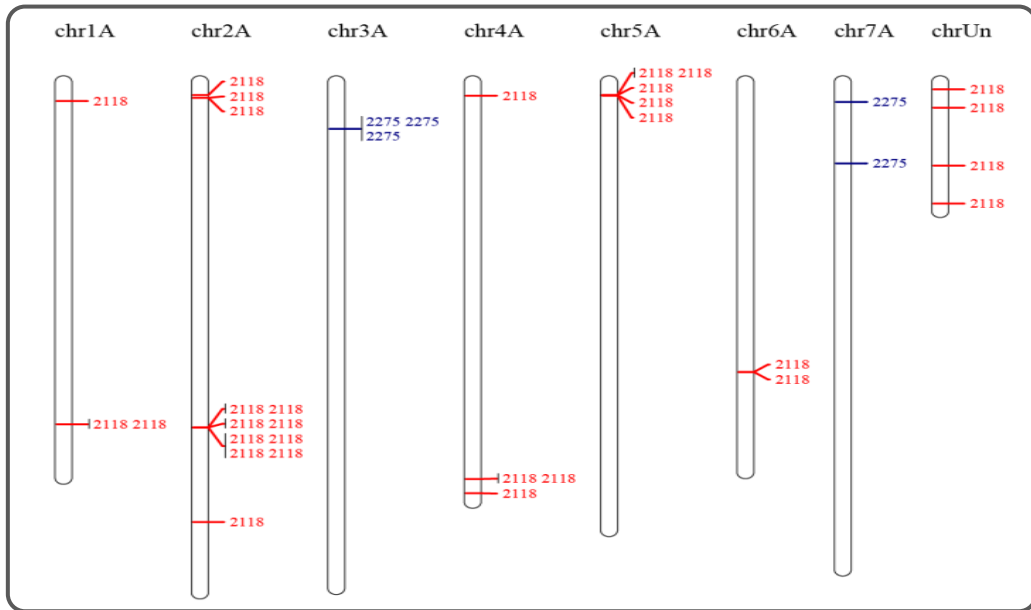

(B)

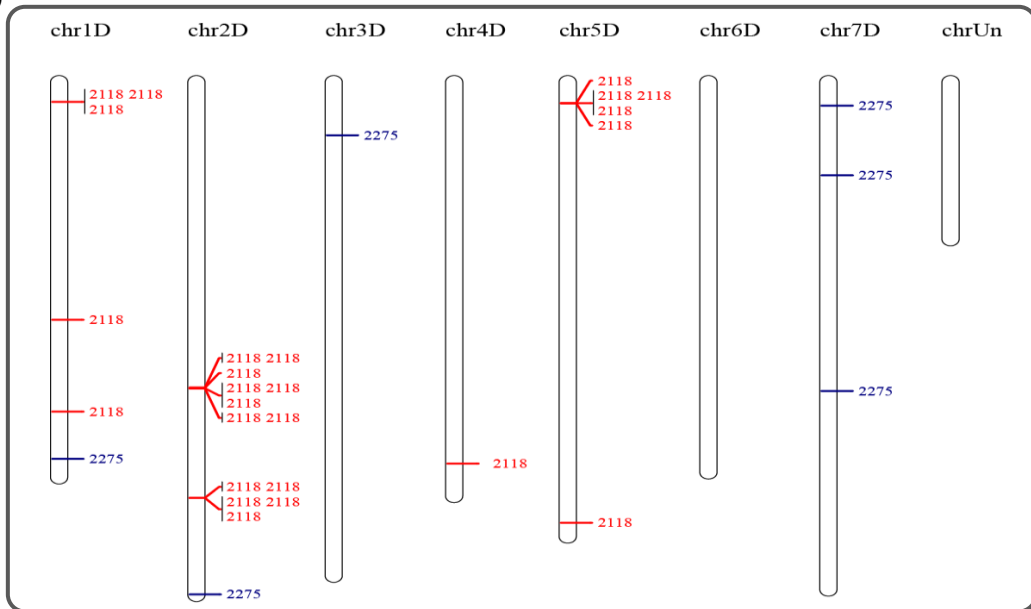

Supplement: Supplementary file 8 — Additional file 8: Supplementary Figure 8. The distribution of miR2118 (red lines) and miR2275 (blue lines) in the AA (A) and DD (B) genomes. [file 12864_2020_6582_MOESM8_ESM.pdf]
