# Supplementary material for: Evolution of PHAS loci in the young spike of Allohexaploid wheat
Source: BMC Genomics. 2020 Mar 4;21:200. doi: 10.1186/s12864-020-6582-4 (PMC7057497; doi:10.1186/s12864-020-6582-4)
Supplement: Supplementary file 3 — Additional file 3: Supplementary Figure 3. The proportions of 21- and 24-PHAS located in gene regions, repeat sequence regions and intergenic regions. [file 12864_2020_6582_MOESM3_ESM.pdf]

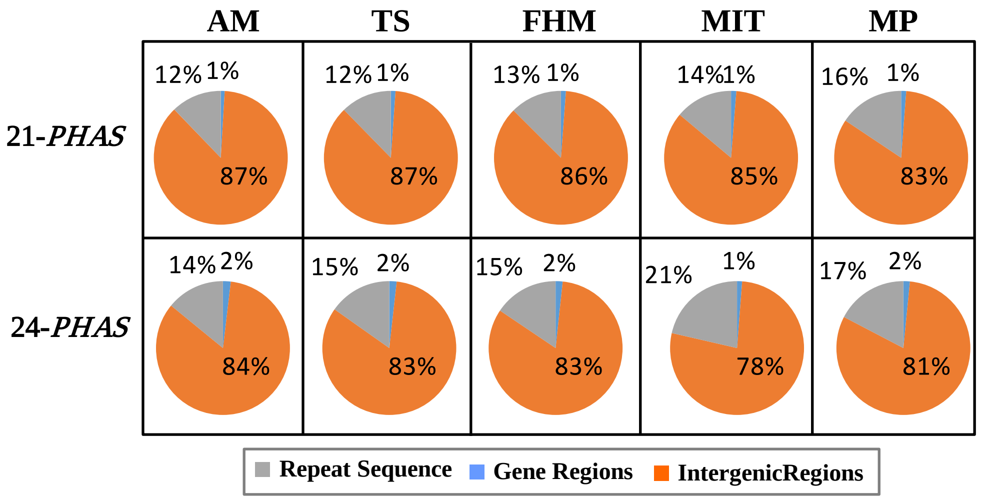

Figure S3. The proportion of 21- and 24-*PHAS* located in gene region, repeat sequence region and intergenic regions.
